# Paratyphoid Fever

**Published:** 1913-11

**Authors:** 


Paratyphoid Fever. Outbreak In Private Surgical Hospital In Roanoke. L. L. Lumsden, Surgeon, U. S. P. H. Service, A. W. Freeman, Asst. State Health Commissioner of Virginia, and W. B. Foster, Health Officer of Roanoke, *Pub. Health Reports,* May 30, 1913. The hospital staff consisted in five physicians, sixteen nurses and eleven servants (32) and thirty-eight patients. Sixteen cases occurred, the first, January 9, in a colored boy employed as helper in the kitchen. The Widal test was negative, but there was clumping with a culture of paratyphoid B. bacilli. The other cases occurred from February 14 to March 26. The incubation period, so far as could be judged from duration of stay in the hospital was from 5-11 days, high fever developed suddenly, reached a maximum of 104 or 105 in two or three days and gradually declined to normal in five days to three weeks, the symptoms being mild, except in one dysenteric case. Rose spots were noted in most of the cases. All but two cases occurred in patients, admitted for operation. There was no epidemic in the town and the community of water, milk and other food supplies, relative lack of insects, etc., left the exact means of conveyance in doubt, though probably it was due to indirect conveyance in a variety of ways. (Note—Aside from the general need of recognition of paratyphoid and of sharply discriminating from true typhoid, "outbreaks" of this nature suggest the need of a technical term as neither epidemic nor endemic is appropriate.)

